# Evaluation of Clinical and Oral Findings in Patients with Epidermolysis bullosa

**DOI:** 10.3390/medicina59071185

**Published:** 2023-06-21

**Authors:** Yasemin Yavuz, Isa An, Betul Yazmaci, Zeki Akkus, Hatice Ortac

**Affiliations:** 1 Restoratif Dentistry, Faculty of Dentistry, Harran University, Şanlıurfa 63000, Turkey; 2 Şanlıurfa Training and Research Hospital, Şanlıurfa 63000, Turkey; 3 Pediatric Dentistry, Faculty of Dentistry, Harran University, Şanlıurfa 63000, Turkey; 4 Faculty of Medicina, Department of Statistic, Dicle University, Diyarbakır 21000, Turkey; 5 Uludag University, Bursa 16000, Turkey

**Keywords:** epidermolysis bullosa, bullae, oral hygiene, dental caries

## Abstract

*Introduction:* Epidermolysis bullosa (EB) is a genetically inherited disease characterized by recurrent bullae and erosions on the skin with numerous signs of dental caries and poor oral hygiene. The aim of this study was to investigate the general clinical and oral findings of patients with EB. *Materials and Methods:* In this prospective study, the clinical and oral findings and family history of 26 cases with EB were evaluated. The type of EB, gender, age, parental consanguinity, dental caries, oral findings, distribution of lesions and presence of associated anomalies, clinical and oral findings correlated with gender were recorded. *Results:* All 26 patients with EB had a history of consanguinity and siblings with EB to varying degrees. In our study, malnutrition, anemia and growth retardation, gastrointestinal system complications, hair thinning, hand and nail deformity, ocular problems and renal disease (in one case) were observed with variable frequencies. When the intraoral findings of the patients were investigated, extensive dental caries in all EB types, enamel hypoplasia in junctional EB (JEB) and the presence of tooth-root to be extracted in dystrophic EB (DEB), intraoral bullae and lesions, ankyloglossia, vestibular sulcus insufficiency, microstomia and maxillary atrophy were observed. Three cases had restorative treatment and one case had prosthetic rehabilitation. *Conclusions:* Oral involvement can be seen with varying frequencies depending on the type of EB and the severity of the disease. It may result from delayed oral and dental rehabilitation due to physical disabilities, limitations and more pressing medical problems. Microstomy, pain from mucosal lesions, and restricted access to the mouth can be caused by poor oral hygiene. Oral complications and caloric needs of individuals with EB should be determined, and individual prophylaxis should be applied to prevent caries formation and protect teeth.

## 1. Introduction

Epidermolysis bullosa (EB) is an inherited disease with 30 different phenotypes and genotypes characterized by mechanical fragility of the skin. The presence of recurrent bullae and erosions on the skin and abnormal wound healing are characteristic features of all types of EB [1]. Problems may be observed on epithelial tissue surfaces, such as gastrointestinal, cardiovascular, genitourinary-system, eye, oral-cavity and dental tissues [2]. EB is caused by mutations in at least 20 different genes encoding various structural and signaling proteins within the epidermis and at the dermis–epidermis junction. These mutations lead to a decrease in the level of proteins that ensure epidermis–dermis integrity and to the development of bullae by the separation of skin layers [3].

EB is classified into four main hereditary types according to the ultrastructural level of bullae within the skin [4]. EB simplex (EBS), junctional EB (JEB), dystrophic EB (DEB) and Kindler EB [1,5]. Subclassification is determined according to clinical phenotypic features, extracutaneous tissue involvement, genetic transmission route and the specific gene affected [5]. Symptoms first appear at birth, but in some patients they may appear in adolescence and later. This may delay making the correct diagnosis until adulthood [6]. There is no approved treatment for EB. The goals in treatment are the prevention of bulla formation, wound care, reduction of pain, early recognition and management of extracutaneous complications [6].

EB is a disease that has multiple oral cavity findings and requires a special treatment approach in terms of oral and dental health.

Although individuals with EB with type VII collagen mutations have a developmentally normal tooth, oral mucosal tissues can be severely affected because they are exposed to oral functions from an early age [7]. Oral lesions are characterized by erythema, blistering and their consequences (e.g., erosion, ulceration, crusting, and atrophic scarring). The number, frequency and severity of lesions depend on the type of disease [7]. Functional sequelae, such as ankyloglossia, microstomy and vestibular sulcus insufficiency, extensive caries, enamel defects and inadequate oral hygiene, require special attention in individuals with EB [7].

EB is a disease that has many oral cavity findings and requires a special treatment approach in terms of oral and dental health. Although individuals with EB with type VII collagen mutations have developmentally normal teeth, their oral mucosal tissues may be severely affected [6]. Dentists are involved in oral treatment planning as part of a multidisciplinary team. Examination of oral soft and hard tissue manifestations of each type of hereditary EB will help in planning long-term treatment approaches [5].

The aim of this study is to investigate the general clinical and oral findings (mucous and dental tissue) of patients with EB and to offer suggestions to help dental health management.

## 2. Materials and Methods

In this prospective study, 26 patients who were clinically, histopathologically and genetically diagnosed with EB, followed up in the dermatology and venereology clinic and consulted to our restorative dentistry and pedodontics clinics between August 2022 and January 2023 were included. Patients’ clinical and oral findings and family history were evaluated. The type of EB, gender, age, parental consanguinity, dental caries, oral findings, lesions and presence of accompanying anomalies were determined and recorded in the detailed anamnesis form.

Ethical approval for this study was obtained from the ethics committee of Harran University Faculty of Medicine (Number: HRU/22.13.08). Informed consent was obtained from all participants and their families. The study was conducted in accordance with the Declaration of Helsinki and Good Clinical Practice guidelines.

In statistical analyses, continuous data were calculated as a mean ± standard deviation (SD) and categorical data were calculated as a frequency (%). Pearson Chi-square test and Fisher’s Exact Test were used to investigate the relationship between general clinical and oral findings and gender. *p* < 0.05 was considered to be statistically relevant. SPSS 25.0 SPSS Inc., PASW Statistics for Windows, Version, 25.0 (Chicago, IL, USA) was used for statistical analysis.

## 3. Results

**Figure 1:**
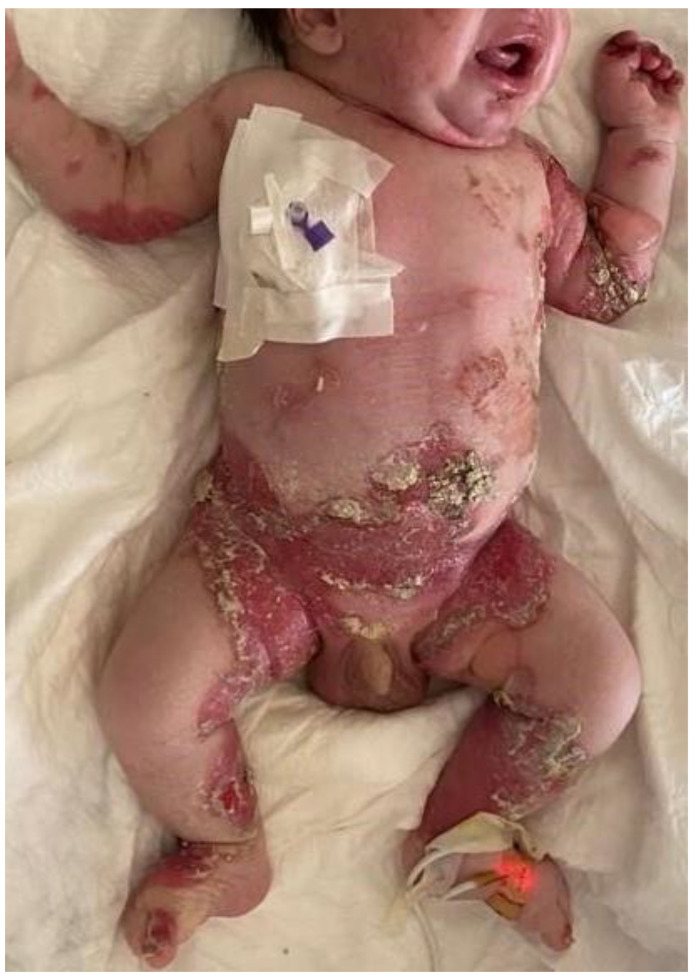
Diffuse bullae and erosion are seen in a patient with a diagnosis of dystrophic epidermolysis bullosa.

Of the 26 patients diagnosed with EB, 11 were female and 15 were male. Of the 26 patients clinically diagnosed with EB, 2 were EBS, 1 was JEB and 23 were DEB (Figure 1). When the relationship between the parents was analyzed, it was found that 100% of the cases had a history of consanguinity to different degrees and 50% of the cases had a sibling with EB.

In our study, malnutrition was observed in 80.7%, anemia in 46%, growth retardation in 61.5%, ocular problems in 42%, various gastrointestinal system complications in 76.9%, hair thinning in 38%, hand and nail deformities in 88% and renal problems in one case. In 26 cases, no hearing problems were observed.

**Figure 2:**
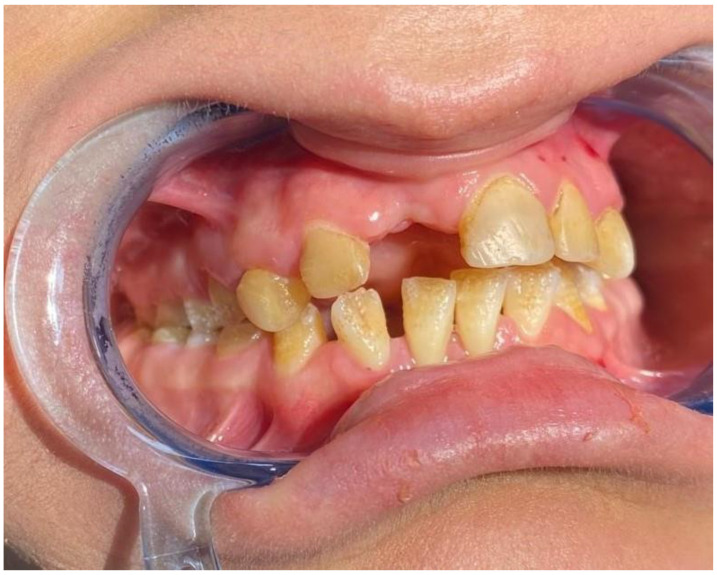
Enamel hypoplasia.

**Figure 3:**
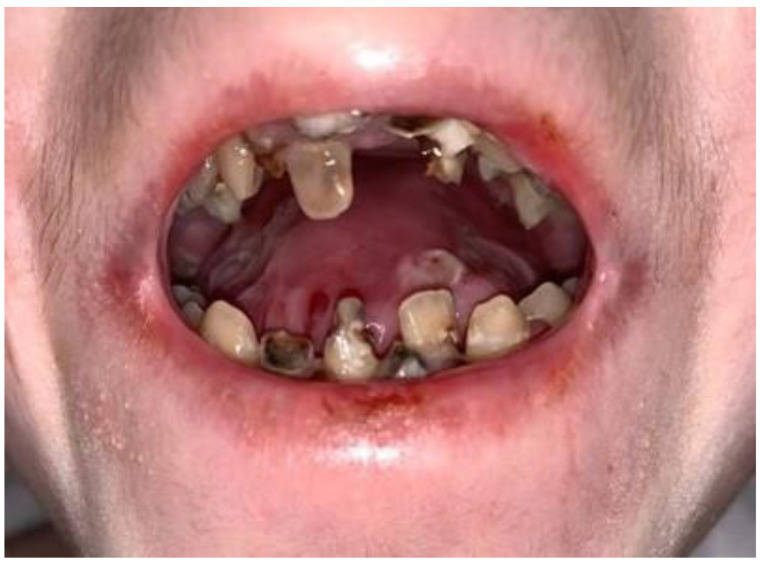
Common dental caries and tooth to be extracted.

**Figure 4:**
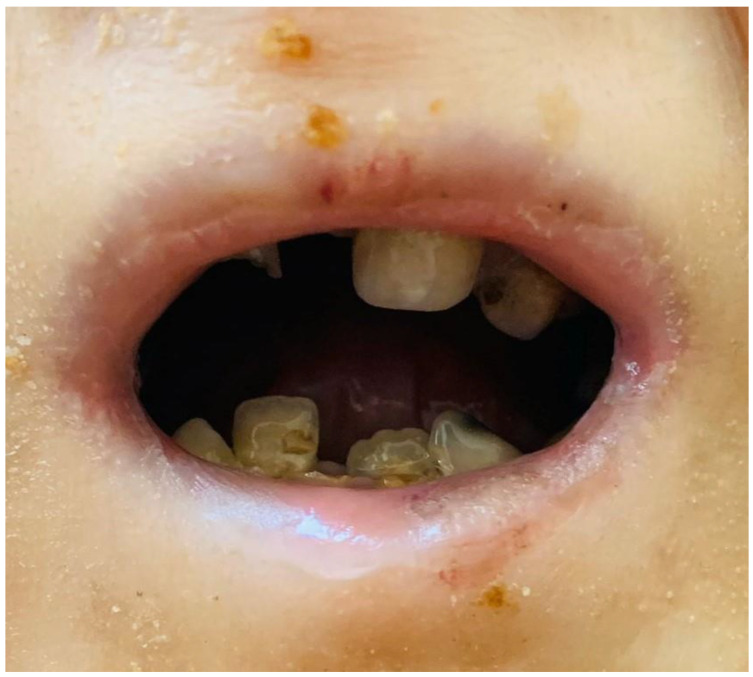
Microstomy.

When the intraoral findings of the patients were investigated, extensive dental caries, enamel hypoplasia in JEB and presence of tooth-root to be extracted in DEB, intraoral bullae and lesions in 92%, ankyloglossia and vestibular sulcus insufficiency in 73%, and microstomia and maxillary atrophy in 69% were observed in all EB types (Figure 2, Figure 3 and Figure 4). In three cases, treated tooth restorations were seen, and in one case, fixed prosthetic zirconium restoration was seen. General clinical findings reported according to EB type are shown in Table 1, and oral findings are shown in Table 2.

## 4. Discussion

EB is a disease caused by genetic defects transmitted in an autosomal dominant or autosomal recessive manner. It has been classified into four main categories according to the topographic location of the bulge within the cutaneous basement membrane [8].

In our study, 88% of individuals with EB had dystrophic variant DEB, one of its four subtypes. DEB is an inherited skin disorder characterized by bullae, erosions and chronic ulcers in the sublamina densa. It has been identified as the COL7A1 gene encoding type VII collagen. In the study conducted by Vahidnezhad et al., COL7A1 mutations in closely related families were compatible with autosomal recessive inheritance [9].

In our study, it was observed that all cases with EB had a history of consanguinity between parents to varying degrees. It was determined that 46% of individuals with EB were siblings. When the gender differences of the affected individuals were examined, no statistically significant difference was observed (*p* > 0.05). The kinship findings we obtained in our study were in parallel with many previous studies that reported that the likelihood of inherited genetic defects in future generations would be higher in societies where consanguineous marriages are common [9,10].

Particular cases of EBS with mild forms of clinical symptoms of EB may not attract the attention of family members or may lead parents to hide such genetic disorders for fear of being ostracized. However, in severe cases of EB, affected individuals are easily included in hospital records [11].

In our study, 88% of EB patients had DEB. DEB patients with mutations in the COL7A1 gene, which encodes type VII collagen, were enrolled in hospital records for their needs.

Nutrition in the first years of an individual’s life is very important for growth and development. Any factor that causes an inadequate nutrient intake during this period predisposes to growth and developmental retardation [12,13].

Manjunath et al. found that dietary modification significantly improved the nutritional status in children with recessive dystrophic epidermolysis bullosa (RDEB) and dominant dystrophic epidermolysis bullosa (DDEB) subtypes. They found that moderate and severe malnutrition in children with EB was significantly associated with the severity of the disease [12].

In our study, all of the developmental delay belonged to the DEB type. Esophageal dilatation was performed in four patients with dysphagia. Growth retardation in individuals with EB can be attributed to various gastrointestinal complications and malnutrition. Dysphagia, esophageal stricture and constipation are the most common gastrointestinal (GI) complications described in DEB [14].

In our study, GI system problems were seen in 76.9% of patients with different severities. It has been reported in previous studies that trauma-induced swelling of the squamous layer of the esophagus following solid food intake and scar tissue formation after healing may cause esophageal strictures [14].

Inadequate nutrient absorption due to bleeding in the GI tract mucosa and cicatrization has been reported to be among the factors causing anemia [15,16].

In our study, growth retardation was observed in 61.5%, malnutrition in 80.7% and anemia in 46% of EB cases. No statistically significant difference was found between EB cases when compared according to the gender variable (*p* > 0.05).

EB is a devastating connective tissue disease that can cause blistering of the ocular surface, including the cornea, conjunctiva and eyelids, poor wound healing and even blindness. Ophthalmic symptoms have been reported to be more common in RDEB caused by VII collagen function and JEB caused by the absence of laminin 5 [17,18].

Chen et al. found that patients with EB showed significant stromal thickening compared to the control group, and visual loss was associated with increased stromal thickness [18].

**Figure 5:**
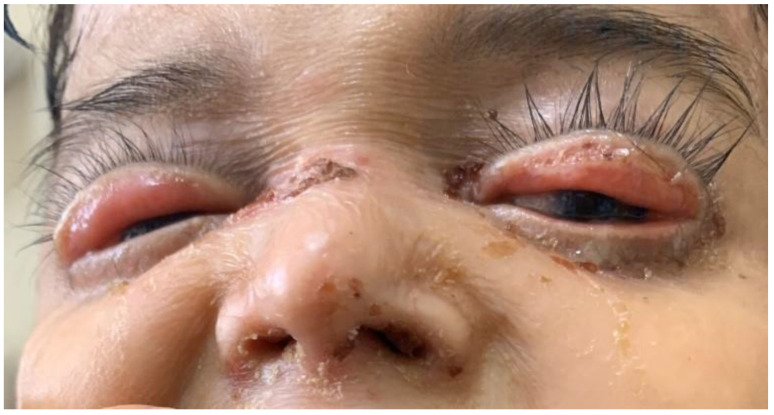
Cicatricial ectropion.

In our study, various ocular problems were seen in 42% of patients. No statistically significant difference was found when these findings were compared according to the gender variable among cases with EB (*p* > 0.05) (Figure 5).

Most patients with DEB have deformities of the fingers and toes of the extremities, and all tissue structures may be affected. Dermal fibrosis, pseudosyndactyly, contractures, atrophic fingers and atrophic thumb tips, nail loss and the entire hand may be covered with an epithelial cocoon [19].

**Figure 6:**
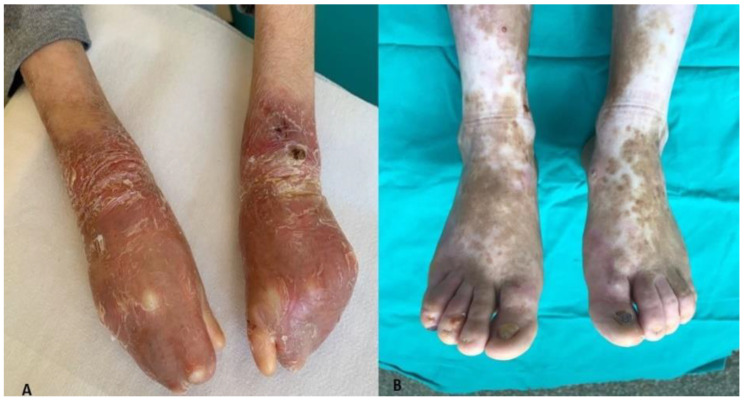
Finger adhesions (**A**) and nail dystrophy (**B**).

Similar findings were observed in our cases. Although the anatomy of the nail differs from that of the skin, the antigenic expression of components of the nail bed, proximal nail fold and basement membrane region of the nail bed is similar to that of normal skin. Although the presence or absence of nail changes is not used as an absolute criterion for a differential diagnosis between the different subtypes of EB, nail dystrophy and loss are seen, especially in JEB and RDEB. Toenails of the big toe are more severely affected due to trauma [20]. In our cases, nail and hand deformities were not seen in EBS, whereas nail dystrophy and absence were present in JEB. Hand and nail deformities were present in all cases of DEB (Figure 6).

No statistically significant difference was found in terms of hand and nail dystrophy when compared according to the gender variable (*p* > 0.05).

Advances in dermal–epidermal-based molecular biology have shown that clinical trials of gene therapy have the potential to make positive changes in the lives of patients with EB [21].

Increased donor cells were found in the skin of children with recessive dystrophic epidermolysis bullosa who underwent allogeneic bone marrow transplantation. Stem cell research may be promising for patients with the most severe forms of EB [22,23].

Oral findings and dental involvement of EB vary according to subtypes. Oral clinical features, such as perioral tissue involvement, microstomia, as well as intraoral soft tissue involvement, such as mouth ulcers, peeled tongue, ankyloglossia, vestibular obliteration, periodontal disease and oral cancer, can be seen. Hard tissue involvement may be accompanied by extensive caries, enamel hypoplasia (local or general), delayed eruption and occlusal anomalies [5,24,25,26].

In our study, intraoral bullae and lesion, ankyloglossia, microstomy, vestibular sulcus insufficiency and maxillary atrophy were observed in all individuals with DEB. In de Azevedo et al.’s study of clinical signs of EB and salivary changes, the RDEB group showed all the features of ankyloglossia in relation to the oral mucosa, and the JEB group showed deformity in the skin and nails. In saliva analysis, the authors observed no difference between the control and EB groups [27].

In terms of dental caries development, all individuals with EB should be managed as high caries risk patients. The high prevalence of caries in individuals with DEB is due to the frequent consumption of high-sugar drinks and soft carbohydrate foods to increase caloric intake in the diet. This means that teeth are in contact with foods with a high cariogenicity for a longer period of time [28,29].

The absence of collagen VII during embryonic development may impair glandular formation. Individuals with EB may have changes in the salivary glands, mammary glands, and skin sebaceous glands [27].

The salivary flow rate, pH and buffering capacity were not found to be different in individuals with EB compared to normal individuals [27,30,31].

In the Prevalence study, it was reported that although the values of all salivary parameters (salivary flow rate, pH and buffering capacity) obtained from individuals with EB were slightly lower than those without EB, there was no significant difference when these parameters were compared with healthy individuals [30].

This suggests that the extensive dental caries seen in EB is most likely attributable to non-salivary factors, such as enamel involvement, soft tissue changes, inadequate oral hygiene and a soft high carbohydrate diet [29,31].

Enamel structural defects (hypoplasia) have been described in EB patients and are associated with EB genetic mutations that also affect ameloblastic differentiation [7]. Individuals with EB caused by mutations in laminin-332 (Lama3, Lamb3, Lamc2), α6ß4-integrin (TGB4, ITGA6) gene and type XVII collagen (Col17A1) are associated with enamel hypoplasia. These proteins are involved in the formation of tooth enamel during amelogenesis [5,6,8].

In our study, developmental enamel defects were seen only in JEB. Although the teeth are structurally normal, inadequate oral hygiene due to microstomia and restricted access to the mouth, pain from mucosal blisters, and a high carbohydrate soft diet may increase the rate of dental caries. No statistically significant difference was found when comparing patients according to the gender variable in terms of dental caries (*p* > 0.05). Wright et al.’s study confirms that the development of caries is not a specific manifestation of EB but rather a consequence of the disruption of oral health-related habits [32].

In their study, Harris et al. reported significantly higher rates of dental caries in children with DEB compared to control groups. Due to difficulty swallowing food, cases with EB tend to eat small amounts of pureed high-carbohydrate foods and drinks throughout the day. This is one of the factors that may explain the high caries experience [29].

In our study, tooth loss was low in all individuals with DEB, but the excess of tooth-root and caries to be extracted was remarkable.

These findings may be explained by the fact that patients primarily focus on vital systemic complications and postpone oral and dental health problems. There are studies reporting that this may be a reflection of the dental treatment needs of dentists and the difficulties in performing restorative treatment [29].

In addition, it has been reported that loss of grasping ability of the fingers due to pseudosyndactyly, difficulty in tooth-brushing, and easy blistering of the oral mucosa in the slightest trauma may negatively affect oral hygiene [29].

In these patients, topical fluoride applications and chlorhexidine mouthwashes may be useful in reducing the burden of cariogenic microorganisms [32].

The oral mucosa originates from ectoderm invagination during the embryologic period. In disorders primarily associated with the skin, mucosal tissues may also be affected. Patients with EB have bullae and ulceration of the oral mucosa. In cases of EBS, bullae tend to be few, to be small in size (<1 cm) and to heal without scarring. Mucosal lesions, which are more common in JEB cases, may heal without obvious scarring, but in severe JEB cases, perioral lesions exhibit granulation tissue. DEB cases often have extremely fragile mucous membranes characterized by tissue fragility. It has been reported that scar-healing ulcerations may have a much higher risk of ankyloglossia, loss of palatal rugae and lingual papillae, vestibule obliteration and microstomia [33].

In our study, no intraoral bullae were observed in EBS cases. The JEB case had intraoral bullae and enamel hypoplasia. Patients with DEB had extensive intraoral soft tissue involvement, including bullae, microstomia, ankyloglossia and obliteration of the oral vestibule. Microstomia, ankyloglossia and vestibular obliteration were not seen in other EB types. When all EB cases were evaluated in terms of microstomia, ankyloglossia and vestibular obliteration according to gender, no statistically significant difference was found (*p* > 0.05).

Mild cases of EB do not need special treatment, but all EB cases should be managed carefully because of their susceptibility to mucosal fragility. Severe forms of EB require special precautions to minimize soft tissue trauma during dental treatments. It has been reported that due to difficulties in oral health care, all EB cases should be encouraged to use fluoride toothpaste, chlorhexidine mouthwash or a spray [29,34,35,36,37,38,39].

## 5. Conclusions

In conclusion, oral involvement can be seen with varying frequencies depending on the type of EB and the severity of the disease:

(1) The presence of extensive caries in individuals with DEB may result from delayed oral and dental rehabilitation due to physical disabilities, limitations and more pressing medical problems.
(2) Microstomy, pain from mucosal lesions, and restricted access to the mouth can be caused by poor oral hygiene.

Nutrition is an important factor for healthy growth and development; therefore, dental treatment needs should be provided without being neglected in the early period. The oral complications and caloric needs of individuals with EB should be determined, and individual prophylaxis should be applied to prevent caries formation and protect teeth.

## Figures and Tables

**Table 1:** Clinical and demographic characteristics of epidermolysis bullosa patients.

		Epidermolysis bullosa Simplex (*n*:2)	Junctional Epidermolysis bullosa (*n*:1)	Dystrophic Epidermolysis bullosa (*n*:23)	Number of Cases (*n*)
Gender	Male	1		14	15
	Female	1	1	9	11
Age (year)		2 and 5	21	0.2–30	26
Parental consanguinity		2	1	23	26
Number of affected siblings		0	0	13	26
Malnutrition		0	0	21	26
Anemia		0	0	12	26
Growth retardation		0	0	16	26
Ocular involvement		0	0	11	26
Auricular involvement		0	0	0	26
Renal involvement		0	0	1	26
Gastrointestinal system involvement		1	1	20	26
Hair involvement		0	1	9	26
Hand and nail deformity		0	1	22	26

**Table 2:** Oral findings in patients with epidermolysis bullosa.

Oral Findings		Epidermolysis bullosa Simplex	Junctional Epidermolysis bullosa	Dystrophic Epidermolysis bullosa	Number of Cases (*n*)
Gender	Male	1		14	15
	Female	1	1	9	11
Age (year)			21	0.2–30	26
Tooth decay		12	10	280	26
Enamel hypoplasia		0	1	0	26
Extracted tooth		0	1	17	26
Tooth to be extracted		0	0	141	26
Periodontal disease		0	1	17	26
Intraoral bulla and erosion		1	1	23	26
Ankyglossia		0	0	19	26
Microstomy		0	0	18	26
Maxillary atrophy		0	0	18	26
Vestibular sulcus insufficiency		0	0	19	26
Restorative treatment history		0	1	2	26
History of prosthetic rehabilitation		0	1	0	26
